# Phase-specific cytotoxicity in vivo of hydroxyurea on murine fibrosarcoma pulmonary nodules.

**DOI:** 10.1038/bjc.1982.72

**Published:** 1982-03

**Authors:** D. J. Grdina

## Abstract

The cytotoxic effects in vivo of hydroxyurea (HU) on murine fibrosarcoma (FSa) cells grown as pulmonary tumours were determined. Tumour cells from 13-day-old nodules were made into suspension and separated on the basis of cell size by centrifugal elutriation. Flow microfluorometry (FMF) was used to determine the cell-cycle parameters and the relative synchrony of the separated populations, as well as the degree of contamination by normal diploid cells in each of the tumour-cell populations. HU cytotoxicity was tested by administering both a single 1 mg/g i.p. dose into mice that had been injected i.v. 20 min earlier with known numbers of synchronized viable FSa cells, and i.p. doses of 1 mg/g each into mice bearing 13-day-old pulmonary nodules. In the latter experiments, animals were killed 1 h after the last dose, and the tumour nodules were excised and made into a single-cell suspension and elutriated. Known numbers of cells from each fraction were injected into recipient mice to determine survival. In both sets of experiments, cell killing by HU correlated with the percentage of S-phase cells. The treatment of 13-day-old pulmonary nodules with 3 doses of HU also depleted the (G2+M) phase tumour cells and increased the heterogeneity between tumour subpopulations, as determined by FMF analysis.


*Br. J. Cancer* (1982) **45**, 438

### PHASE-SPECIFIC CYTOTOXICITY *IN VIVO* OF HYDROXYUREA ON MURINE FIBROSARCOMA PULMONARY NODULES

D. J. GRDINA

*From the Department of Experimental Radiotherapy, M. D. Anderson Hospital and Tumor Institute, Houston, Texas 77030, U.S.A.*

Received 3 August 1981 Accepted 12 November 1981

**Summary.—The cytotoxic effects *in vivo* of hydroxyurea (HU) on murine fibrosarcoma (FSa) cells grown as pulmonary tumours were determined. Tumour cells from 13-day-old nodules were made into suspension and separated on the basis of cell size by centrifugal elutriation. Flow microfluorometry (FMF) was used to determine the cell-cycle parameters and the relative synchrony of the separated populations, as well as the degree of contamination by normal diploid cells in each of the tumour-cell populations. HU cytotoxicity was tested by administering both a single 1mg/g i.p. dose into mice that had been injected i.v. 20 min earlier with known numbers of synchronized viable FSa cells, and i.p. doses of 1 mg/g each into mice bearing 13-day-old pulmonary nodules. In the latter experiments, animals were killed 1 h after the last dose, and the tumour nodules were excised and made into a single-cell suspension and elutriated. Known numbers of cells from each fraction were injected into recipient mice to determine survival. In both sets of experiments, cell killing by HU correlated with the percentage of S-phase cells. The treatment of 13-day-old pulmonary nodules with 3 doses of HU also depleted the ($G_2$+M) phase tumour cells and increased the heterogeneity between tumour subpopulations, as determined by FMF analysis.**

MANY OF THE CHEMICAL AGENTS currently used in cancer therapy have phase-specific cytotoxicity. To evaluate these agents better before clinical trials, it would be advantageous to characterize their effectiveness on synchronized target cells within living animals. Recently, an *in vivo* method of studying the cell-cycle phase specific effects of a variety of chemotherapeutic agents *in vivo* was described (Grdina *et al.*, 1979, 1980). Target tumour populations, enriched with cells in either $G_1$, S or $G_2$+M phase by centrifugal elutriation, were injected i.v. into mice 20 min before the i.v., i.p., or s.c. administration of the drug to be tested. With the appropriate controls, the phase-specific cytotoxicity of any test agent could be determined (Grdina *et al.*, 1979). A limitation of this procedure, however, was the need for the inherent heterogeneity of the tumour-cell populations derived from solid tumours to be reduced for cell separation by centrifugal elutriation to be effective (Anderson *et al.*, 1969; Grdina *et al.*, 1978*b*). This was accomplished by a 48h *in vitro* incubation (Grdina *et al.*, 1978*a*). To avoid this limitation, the method of centrifugal elutriation has been applied to separate murine fibrosarcoma (FSa) cells from pulmonary tumour nodules grown in C3H mice. Hydroxyurea (HU), because of its well characterized S-phase-specific cytotoxicity (Kim *et al.*, 1967; Sinclair, 1967), was chosen to demonstrate the applicability of this procedure to characterizing the *in vivo* effectiveness of phasespecific chemotherapeutic agents on advanced metastatic disease.

### MATERIALS AND METHODS

*Mice and tumour.*—Female C3Hf/Kam mice, 10–12 weeks old, from our specificpathogen-free breeding colony and a methycholanthrene-induced fibrosarcoma were used (Suit & Suchato, 1967). Tumours, 6th-generation isotransplants, were made into viable single-cell suspensions and injected into untreated recipient mice to produce 100–150 pulmonary nodules (Grdina *et al.* 1978*b*). After 13 days, tumour-bearing animals were killed, either immediately or after treatment with multiple doses of HU. Tumour cells derived from these pulmonary nodules were used in all the experiments.

*Tumour-cell suspension.*—Single-cell suspensions were prepared by mincing and trypsinizing colonies of tumour cells from excised lungs (Grdina *et al.*, 1979). Because no advantage was found from the careful excision of individual tumour nodules from surrounding lung tissue, cell suspensions were routinely made from entire lung lobes containing tumour tissue. Briefly, lung lobes, separated and removed from the surrounding viscera, were carefully minced with ophthalmic scissors. The mince, containing normal and tumour tissue, was added to a beaker containing 0·025% trypsin in Solution A (8·0 g NaCl, 0·4 g KCl, 1·0 g glucose and 0·35 g $NaHCO_3$ in a litre of water) and stirred on a magnetic stirrer for 20 min at room temperature. DNase (crude Deoxyribonuclease 1 from beef pancreas; Sigma Chemical Co., St Louis, MO) was also added to the mixture to achieve a final concentration of 0·1 mg/ml. After stirring, the beaker was allowed to stand undisturbed for 5 min. Highly enriched undigested tumour tissue settled to the bottom of the beaker, whereas most of the lung tissue floated to the top. While exercising care to avoid collecting floating lung tissue, the upper two-thirds of the suspension was removed and mixed with an equal volume of a modified McCoy's 5A growth medium supplemented with 20% foetal calf serum (Humphrey *et al.*, 1970). The stirring and collection procedure was repeated ×3. Each resultant suspension was passed through a stainless-steel mesh (200 wires/inch) and centrifuged at 225 *g* for 5 min. The supernatants were discarded, and the pellets were resuspended in McCoy's 5A supplemented with 5% FCS containing DNase at a final concentration of 0·1 mg/ml. Also included was 5mM 2-napthol 6-8 disulphonic acid to reduce cell clumping (Shortman, 1973). After centrifugal elutriation, cell viability was routinely >95% as determined by phase-contrast microscopy.

*Separation by centrifugal elutriation.*—FSa cells derived from pulmonary nodules were separated under sterile conditions by centrifugal elutriation using the same procedure described for their separation from either solid tumours or tissue culture (Grdina *et al.*, 1978*a*, 1979). With the rotor speed set at 1525 rev/min, $2\text{–}3 \times 10^8$ cells, suspended in 20 ml of medium, were introduced into the elutriator chamber at a flow rate of 5·4 ml/min. The rotor speed was held constant throughout the separation, and the flow rates were varied by equal increments from 5·4 to 27·4 ml/min. Routinely, 12 fractions (F), each of 50 ml, were collected and stored at 4°C. Cells collected in each fraction were counted by haemacytometer and by Coulter Counter (model ZB1; Coulter Electronics, Hialeah, FL), and their volume distributions determined with a multichannel analyser (Channelyzer II; Coulter Electronics). The modal volume was designated as that corresponding to the modal channel number of the volume distribution of each sample (Grdina *et al.*, 1978*a*). F 1 containing small cells and cellular debris, and F 11 and 12, containing a heterogeneous mixture of cells, were discarded.

*Flow microfluorometry.*—The DNA content of individual cells in suspension was determined by FMF using an ICP 11 flow cytometer (Phywe Co., Gottingen, Germany). cells were fixed in 70% ethanol and then stained with 50 mg/ml mithramycin (Mithracin; Pfizer and Co., Inc., New York, NY) in solution with $MgCl_2$ (7·5 mM) and 12·5% aqueous ethanol (Grdina *et al.*, 1978*a*). The resultant histograms of DNA fluorescence were computer-analysed (Johnston *et al.*, 1978). Because cell suspensions were derived directly from FSa lung nodules, a considerable number of normal diploid lung cells were present in all the elutriator fractions. Since FSa cells are heteroploid (*i.e.*, 60–70 chromosomes) and contain ~1·8 as much DNA as normal diploid cells (Grdina *et al.*, 1977), an estimate of the normal cell contamination in each of the tumour-cell suspensions can be made by determining the area under the $G_1$ normal diploid peak and dividing it by the area under the total FMF histogram (*i.e.*, the area under both the tumour and normal peaks) (Grdina *et al.*, 1978*a*). While the relative proportions of normal cells in the S and $G_2+M$ phases contaminating FSa tumour-cell suspensions are uncertain (*i.e.*, fluorescence from these cells would be detected at the same channels as those from $G_1$ tumour cells), they are considered to be sufficiently rare in this experimental system to be ignored in these calculations. FMF histograms of lung cells from tumour-free animals indicate that less than 2% of the cells are in S or $G_2+M$ phases (unpublished data). Likewise, few if any diploid cells appear to be in the S phase in suspensions from pulmonary nodules (*i.e.*, low background fluorescence in Channels 60 to 80, see Figs. 2 & 6). Therefore all cell counts were adjusted after FMF analysis to represent only tumour-cell numbers.

*Lung colony assay.*—The colony-forming efficiency (CFE) of FSa cells was determined in a lung colony assay. Recipient mice, with their hind legs shielded, were whole-body-irradiated with 10 Gy 24 h before use. These mice were then injected with known numbers of viable FSa cells, corrected for normal-cell contamination, from each of the elutriator fractions and the unseparated control (USC) population. Each aliquot of cells also contained $2 \times 10^6$ heavily irradiated (HIR; 100 Gy) FSa cells. HIR cells were not separated by centrifugal elutriation. Thirteen days alter the mice were killed, their lungs were removed, and the lobes separated and fixed in Bouin's solution, and tumour colonies counted.

*HU testing* in vivo.—HU (manufactured by Ben Venue Laboratories, Bedford, OH) was obtained from the Division of Cancer Treatment, National Cancer Institute, National Institutes of Health, Bethesda, MD. Stock solutions were made up at 100 mg/ml in sterile water. Two procedures, designated A and B, were followed to test HU's effectiveness *in vivo.* At least 3 replicate experiments were performed using each procedure. In procedure A, FSa cells grown as pulmonary nodules were harvested, made into suspension, and separated by centrifugal elutriation before drug treatment according to the method described earlier (Grdina *et al.*, 1979). Twenty minutes after the injection of viable synchronized FSa cells, 10 of the 20 animals in each group were injected i.p. with a single dose of HU at 1·0 mg/g body wt. Thirteen days later the animals were killed, and the resulting colonies were counted. In Procedure B, mice bearing 13-day-old pulmonary nodules were injected i.p. each hour with 1 mg/g HU for 3 h (*i.e.*, total dose 3 mg/g). One hour after the last injection, the animals were killed and their lungs removed. Suspensions of tumour cells were made and separated by centrifugal elutriation. The CFE of these cells was determined in a lung-colony assay with preconditioned mice.

### RESULTS

The recovery of cells from centrifugal elutriation was routinely >90% and the viability of these cells, determined by phase-contrast microscopy, was >95%. Fig. 1 shows a representative sedimentation profile of the relationship between modal cell volume and the number of cells recovered in each elutriator fraction. Analysis of the DNA histograms for each separated population enabled the distribution of tumour cells with respect to DNA content to be determined (Fig. 2). The average sedimentation velocity of

FIG. 1.—Separation of FSa tumour cells from lung metastasis by centrifugal elutriation. The percentage of total cells (●——●), the FMF corrected percentage of tumour cells only (○— —○), and the modal cell volume (▲— —▲) are plotted as a function of sedimentation velocity and fraction number.

FIG. 2—Representative DNA histograms obtained by FMF analysis of an unseparated lung and tumour-cell suspension (USC) and fractions of cells separated from that suspension by centrifugal elutriation ($F_2-F_{10}$). Normal diploid $G_1 = G_1(N)$, tumour $G_1 = G_1(T)$; and tumour $G_2 = G_2 + M(T)$.

FIG. 3.—The colony-forming efficiency (CFE) of FSa cells separated by centrifugal elutriation from lung metastasis. The CFE uncorrected (▲——▲) and corrected (○——○) for contaminating normal diploid cells (as determined by FMF analysis) is plotted as a function of fraction number. Each vertical bar represents ± s.e.

these cells was 11·4 mm/h/g, and their average modal volume was 1250 $\mu m^3$. The percentage of normal diploid $G_1$ cells and tumour $G_1$, S and ($G_2$+M) cells, and the coefficients of variation (CV) of the tumour $G_1$ fluorescence peaks, as calculated from the histograms in Fig. 2, are presented in Table I. In contrast to results with tumour cells cultured *in vitro* for 48 h (Grdina *et al.*, 1979) normal cell populations were observed in all elutriator fractions, with the largest percentage of normal diploid cells in F 2.

The CFE of recovered cells, both uncorrected and corrected for normal diploid cell contamination by FMF analysis, is presented in Fig. 3. The average modal volumes of cells collected in F 2 and 3 were < 800 $\mu m^3$. The CFE of tumour cells collected in these fractions, even after FMF correction for diploid cells, was significantly less than for cells in the other fractions.

TABLE I.—*Distribution of untreated cells in various phases of the cell cycle after centrifugal elutriation (Determined by FMF analysis)*

| Fraction Number | % Cellls in | | | | |
|---|---|---|---|---|---|
| | $G_1$(N)* | $G_1$(T)† | S(T) | G2+M(T) | CV of $G_1$(T) peak |
| USC | 21 | 65 | 18 | 17 | 4·1 |
| 2 | 46 | 91 | 9 | 0 | 3·2 |
| 3 | 26 | 86 | 11 | 3 | 1·4 |
| 4 | 14 | 81 | 16 | 3 | 3·8 |
| 5 | 13 | 73 | 24 | 3 | 4·0 |
| 6 | 10 | 27 | 43 | 30 | 4·4 |
| 7 | 15 | 17 | 37 | 46 | 4·5 |
| 8 | 15 | 16 | 19 | 65 | 4·6 |
| 9 | 14 | 12 | 17 | 71 | 6·0 |
| 10 | 12 | 18 | 16 | 66 | 5·8 |

* $G_1$ of normal diploid cells.

† $G_1$ of tumour cells.

The cell-killing of a single dose of HU on FSa cells lodged in the lungs of test animals (*i.e.*, Procedure A) is presented in Fig. 4. The CFE of both the control and treated populations was routinely corrected for normal-diploid-cell contamination. Cell killing with HU was most evident for FSa cells collected in F 6 and 7. These fractions contained the greatest concentrations of cells with S-phase DNA content. These data are consistent with the results using cultured FSa cells (Grdina *et al.*, 1979).

The cytotoxicity of 3 equal *in vivo* doses of HU at hourly intervals to FSa lung-nodule cells (*i.e.*, Procedure B) is shown in Fig. 5. Animals were killed 1 h after the last dose, lungs were removed and made into a single-cell suspension, and cells were separated by centrifugal elutriation. DNA histograms describing the separated tumour populations following HU treatment are presented in Fig. 6. Whereas the fluorescence peaks representing normal diploid $G_1$ cells remained unperturbed and comparable to those of untreated populations (Fig. 2), those representing HU-treated $G_1$ and ($G_2$+M) tumour cells are broader and more heterogeneous. Nevertheless, cell killing again correlated with the percentage of cells in S (see Fig. 5). A significant reduction in the percentage of cells with ($G_2$+M) DNA content was also found after this treatment, which is not surprising since the total treatment time was 4 h, and the duration of $G_2$+M FSa cells *in vivo* is only 2·8 h (Grdina, 1982). The percentages of normal diploid $G_1$ and tumour $G_1$, S, and ($G_2$+M) cells, and the CVs of the $G_1$ tumour fluorescence peaks are contained in Table II.

### DISCUSSION

Although centrifugal elutriation has been successfully used to separate and isolate populations of FSa tumour cells enriched in $G_1$, S or ($G_2$+M) phases following growth *in vitro* (Grdina *et al.*, 1978*b*, 1979) it has not been effective for synchronizing FSa cells derived directly from solid tumours growing *in vivo* (Grdina *et al.*, 1977, 1978*b*). The cellular parameter that is exploited using this procedure is cell volume, because the sedimentation rate of a cell is proportional to the two-thirds power of its volume (Glick *et al.*, 1971). Cell size, under conditions of uniform growth, is known to increase during the division cycle (Anderson *et al.*, 1969). Thus, after exponential growth *in vitro*, the relationship between cell size and DNA content is readily exploitable for FSa tumour cells. In contrast, cells growing in solid tumours are exposed to a variety of physiological conditions, including variations in the availability of nutrients, $O_2$ tension and pH. Consequently tumour cells of various sizes can have

FIG. 4.—The percentage of surviving FSa cells after exposure *in vivo* as single cells to 1mg/g hydroxyurea (HU) (see Procedure A) above and the percentage of cells distributed among the various cell-cycle phases (below) each plotted as a function of fraction number. ●, $G_1$; ▲, S; □, $G_2$+M. Data are presented from a representative experiment and each vertical bar represents ± s.e.

FIG. 5.—The percentage of surviving FSa cells after exposure *in vivo* as 13-day-old lung nodules to 3 doses of hydroxyurea (HU) (see Procedure B) (above) and the percentage of cells distributed among the various cell-cycle phases (below) are each plotted as a function of fraction number. Data are from a representative experiment. Symbols as in Fig. 4.

similar DNA contents, making separation of cell populations on the basis of cell size ineffective (Sigdestad & Grdina, 1981). Although FSa cell populations from pulmonary nodules are also more heterogeneous than those from exponentially growing cultures *in vitro*, they are considerably less so than those from solid tumours, which is not surprising since the variations in the microenvironment in solid tumours are, presumably, considerably greater than those in the smaller lung-tumour nodules. Biological parameters that appear to reflect this "intermediate" situation include a modal cell volume of 1250 $\mu m^3$ and an average sedimentation velocity of 11·4 mm/h/g for FSa cells from pulmonary nodules, compared to modal volumes of 980 and 1620 $\mu m^3$ and sedimentation velocities of 10·7 and 15·1 mm/h/g for FSa cells from solid tumours and *in vitro* cultures, respectively (Grdina *et al.*, 1978*a*). These differences cannot be explained solely by variations in the cell-cycle distribution of each of these populations; since FSa cell suspensions from pulmonary nodules and solid tumours exhibit similar DNA histograms by FMF analysis (Grdina *et al.*, 1977, 1978*a*).

Little if any variation in CFE or contamination with normal cells was

FIG. 6.—Representative DNA histograms obtained by FMF analysis of an unseparated tumour-cell suspension (USC) and fractions of cells separated from that suspension by centrifugal elutriation ($F_2 - F_{10}$) following *in vivo* exposure to 3 doses of HU.

evident for FSa cells collected in elutriator F 4–10. Tumour cells in the first 3 fractions, however, had a significantly reduced CFE, even after correction for normal cell contamination. The modal volume for cells in each of these fractions was $< 800\ \mu m^3$. The reduced CFE of these populations is consistent with results published in an earlier report on the reduced CFE of small (*i.e.*, $< 800\ \mu m^3$) FSa cells from solid tumours (Grdina *et al.*, 1978*b*). This may reflect a reduced intrinsic proliferative ability of these cells or a less efficient retention of cells in the lungs. However, in similar studies with L-P59 sarcoma cells assayed *in vitro*, the smallest tumour cells had significantly low CFE, suggesting that small tumour cells (*i.e.*, $< 800\ \mu m^3$) separated by centrifugal elutriation are intrinsically the least clonogenic (Meistrich *et al.*, 1977).

Because FSa cells were separated from tumour nodules growing in murine lung tissue, tumour cell suspensions were always contaminated by normal diploid lung cells. Attempts to extract individual lung nodules before the preparation of cell suspensions in order to reduce this contamination substantially were unsuccessful. After centrifugal elutriation, however, 65–85% of the normal diploid cells in the initial cell suspension, as determined by FMF analysis, were routinely collected in the first 3 fractions. This procedure may therefore prove to be an effective means of enriching the proportion of

TABLE II.—*Distribution of HU-Treated* (3/1/*mg*/*g*) *cells in various phases of the cell cycle after centrifugal elutriation* (*Determined by FMF analysis*)

| Fraction number | % Cells in $G_1(N)$ | $G_1(T)$ | S(T) | $G^2+M(T)$ | CV of $G_1(T)$ peak |
|---|---|---|---|---|---|
| USC | 36 | 73 | 22 | 5 | 6·2 |
| 2 | 71 | 94 | 6 | 0 | 5·7 |
| 3 | 59 | 86 | 11 | 3 | 6·0 |
| 4 | 13 | 84 | 11 | 5 | 5·2 |
| 5 | 14 | 77 | 20 | 3 | 5·3 |
| 6 | 15 | 65 | 27 | 8 | 5·8 |
| 7 | 18 | 52 | 34 | 14 | 7·4 |
| 8 | 22 | 37 | 48 | 15 | 6·3 |
| 9 | 28 | 29 | 55 | 16 | 7·1 |
| 10 | 24 | 17 | 56 | 17 | 8·0 |

malignant cells in tumour-cell suspensions. Studies on the effect(s) of host cells on tumour growth and kinetics might be facilitated in this way.

FSa cells, synchronized by centrifugal elutriation following growth as pulmonary nodules and injected into recipient mice (*i.e.*, Procedure A), responded to a single i.p. dose of HU in a manner similar to that described for FSa cells separated from exponential cultures (Grdina *et al.*, 1979). HU was administered 20 min after tumour-cell injection in this procedure, because at this time $>95\%$ of the injected cells are retained in the lungs (Grdina *et al.*, 1978*b*). As described in an earlier report, cell killing by HU was strongly correlated with the percentage of cells in S phase, as determined by FMF analysis (Fig. 4).

The use of Procedure A for assessing the cycle-dependence of cytotoxic agents *in vivo* is predicated upon direct cell killing of target tumour cells. A phenomenon that could preclude the usefulness of this method would be a drug-mediated effect on the host response to the injected tumour cells that might selectively (*i.e.*, as a function of their position within the division cycle), affect their clonogenic capacity. Two extremely effective agents that are known to enhance CFE of injected tumour cells are radiation and cyclophosphamide. However, using mice preconditioned with either of these agents, both lung-retention patterns of $^{125}$I-labelled FSa cells and the CFE of unlabelled cells were found not to vary with either cell size or position within the division cycle (Grdina *et al.*, 1978*b*). Thus, under these conditions Procedure A remains an effective method for characterizing the relative *in vivo* phase specificity of cytotoxic agents such as HU. The usefulness of this procedure is confirmed by the close agreement between data acquired in this manner and those derived from established *in vitro* methods (Grdina *et al.*, 1980; Mehn *et al.*, 1980).

The S-phase-specific cytotoxicity of HU to FSa cells was also demonstrable after *in situ* treatment of 13-day-old tumour lung nodules (*i.e.*, Procedure B; Fig. 6). Cell killing was again correlated with the fraction of cells in S phase. No reduction in cell recovery was evident, either in the preparation of cell suspensions or by centrifugal elutriation. Because the treatment time exceeded the duration of $G_2+M$ (Grdina, 1982) it was not surprising that the proportion of cells in this phase was diminished. FMF analysis was made difficult, however, by the bimodal nature of the fluorescence peaks describing the DNA distributions of the tumour populations (Fig. 6). This heterogeneity may have been due to a differential effect of HU on either the progression and killing and/or the stainability by mithramycin of cells in at least 2 distinct classes (*i.e.*, with respect to DNA content) of FSa cells. Because of the relatively short exposure, it is unlikely that HU altered the DNA content of FSa cells. Rather, it is more likely that HU acted somehow to affect the stainability of a class of FSa cells by mithramycin. This phenomenon has been described in Chinese hamster ovary cells treated with bromodeoxyuridine(Swartzendruber, 1977). It is also interesting that the fluorescence peak describing the normal-diploid cells from each elutriator fraction was not affected by this treatment.

In conclusion, the phase-specific cytotoxicity of HU, administered in either a single- or multiple-dose schedule, on FSa cells from pulmonary nodules was described. In addition to measuring cytotoxicity, this procedure can be used to monitor and characterize perturbations in cell kinetics, both during and after therapeutic treatment. Target tumour systems can be single cells lodged in the lungs as well as pulmonary nodules of various ages and sizes. In this manner, various therapeutic modalities used either alone or in combination can be routinely and rapidly tested and characterized under *in vivo* conditions.

This work was conducted with the excellent technical assistance of Sandra Jones, Nancy Hunter,

Jean Jovonovich, and Gary Zin. We thank Dr B. Barlogie for supplying us with the DNA-specific mithramycin, and Dr A. White for helping us with the computer analysis of the data. In addition, we are grateful to Debbie Palmatary and her staff for the supply and care of the animals used in these experiments. This investigation was supported in part by grants numbered CA-18628, CA-23270, and CA-06294, awarded by the National Cancer Institute, DHEW.

### REFERENCES

Anderson, E. C., Bell, G. I., Peterson, D. F. & Tobey, R. A. (1969) Cell growth and division. IV. Determination of volume growth rate and division probability. *Biophys. J.*, **9**, 246.

Glick, D., Yuhas, V. E. & McEwen, C. R. (1971) Separation of mast cells by centrifugal elutriation. *Exp. Cell Res.*, **65**, 23.

Grdina, D. J., Hittelman, W. N., White, R. A. & Meistrich, M. L. (1977) Relevance of density, size and DNA content of tumour cells to the lung colony assay. *Br. J. Cancer*, **36**, 659.

Grdina, D. J., Peters, L. J., Jones, S. & Chan, E. (1978*a*) Separation of cells from a murine fibrosarcoma on the basis of size. I. Relationship between cell size and age as modified by growth *in vivo* or *in vitro*. *J. Natl Cancer Inst.*, **61**, 209.

Grdina, D. J., Peters, L. J., Jones, S. & Chan, E. (1978*b*) Separation of cells from a murine fibrosarcoma on the basis of size. II. Differential effects of cell size and age on lung retention and colony formation in normal and pre-conditioned mice. *J. Natl. Cancer Inst.*, **61**, 215.

Grdina, D. J., Sigdestad, C. P. & Peters, L. J. (1979) Phase-specific cytotoxicity *in vivo* of hydroxyurea on murine fibrosarcoma cells synchronized by centrifugal elutriation. *Br. J. Cancer.*, **39**, 152.

Grdina, D. J., Sigdestad, C. P. & Peters, L. J. (1980) Cytotoxic effect *in vivo* of selected chemotherapeutic agents on synchronized murine fibrosarcoma cells. *Br. J. Cancer.* **42**, 677.

Grdina, D. J. (1982) Cell separation techniques as applied to the study of a murine fibrosarcoma. In *Methods in Tumour Biology: Tissue Culture and Clinical Models*, (Ed. Sridhar) New York: Marcel Decker, (in press).

Humphrey, R., Steward, D. & Sedita, B. (1970) DNA strand scission and rejoining in mammalian cells. In *Genetic Concepts and Neoplasia*, Baltimore: Williams and Wilkins, p. 570.

Johnston, D. A., White, R. A. & Barlogie, B. (1978) Automatic processing and interpretation of DNA distributions: Comparison of several techniques. *Comput. Biomed. Res.*, **11**, 393.

Kim, J. H., Gelbard, A. S. & Perez, A. G. (1967) Action of hydroxyurea on the nucleic acid metabolism and viability of HeLa cells. *Cancer Res.*, **27**, 1301.

Meistrich, M. L., Grdina, D. J., Meyn, R. E. & Barlogie, B. (1977) Separation of cells from mouse solid tumors by centrifugal elutriation. *Cancer Res.*, **37**, 4291.

Meyn, R. E., Meistrich, M. L. & White, R. A. (1980) Cycle-dependent anticancer drug cytotoxicity in mammalian cells synchronized by centrifugal elutriation. *J. Natl Cancer Inst.*, **64**, 1215.

Shortman, K. (1973) Physical procedures for the separation of animal cells. *Ann. Rev. Biophys. Bioeng.*, **7**, 93.

Sigdestad, C. P. & Grdina, D. J. (1981) Density centrifugation of murine fibrosarcoma cells following *in situ* labelling with tritiated thymidine. *Cell Tissue Kinet.*, **14**, 589.

Sinclair, W. K. (1967) Hydroxyurea: Effects on Chinese hamster cells grown in culture. *Cancer Res.*, **27**, 297.

Suit, H. D. & Suchato, D. (1967) Hyperbaric oxygen and radiotherapy of fibrosarcoma and of squamous-cell carcinoma. *Radiology*, **89**, 713.

Swartzendruber, D. E. (1977) A bromodeoxyuridine (BUdR)-mithramycin technique for detecting cycling and non-cycling cells by flow microfluorometry. *Exp. Cell Res.*, **109**, 439.


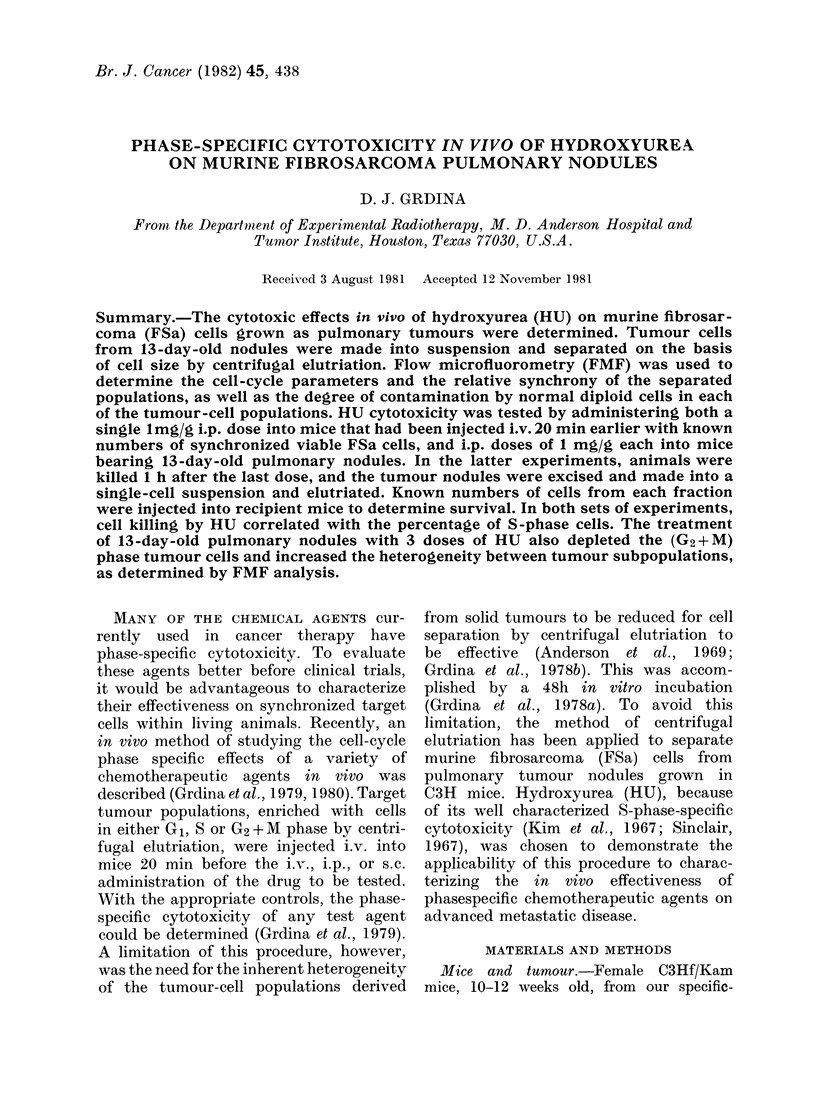

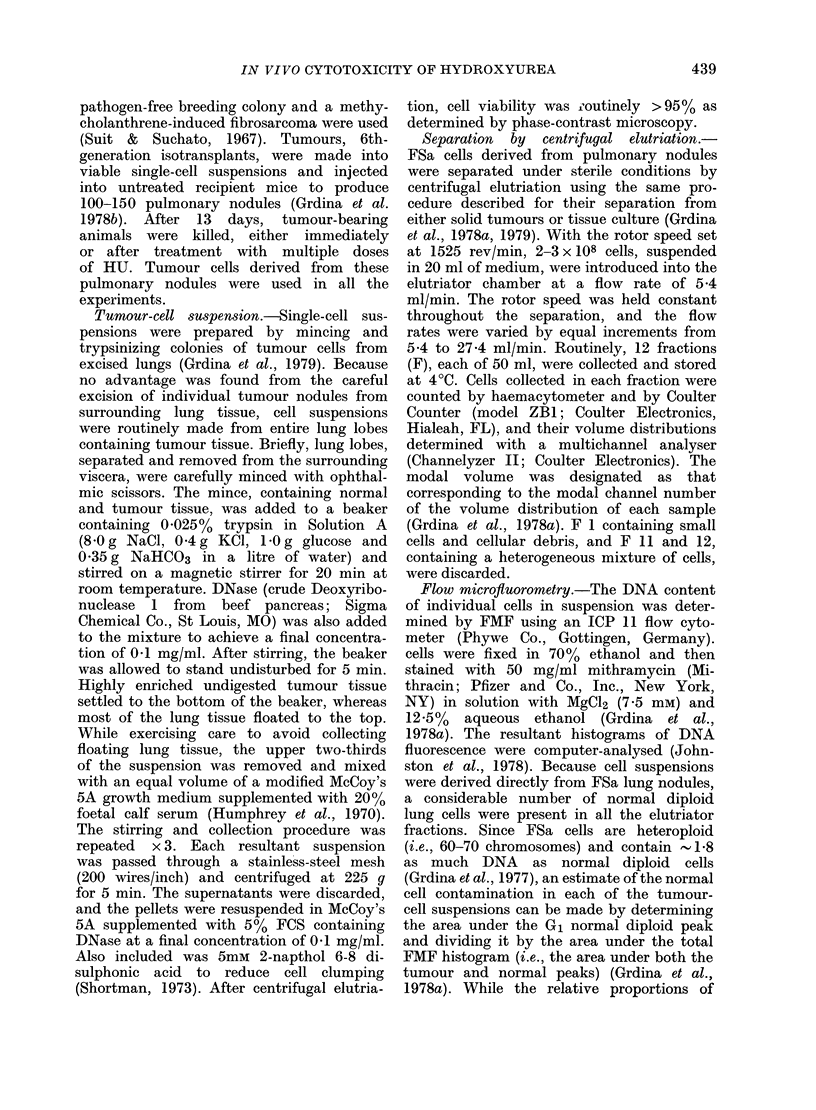

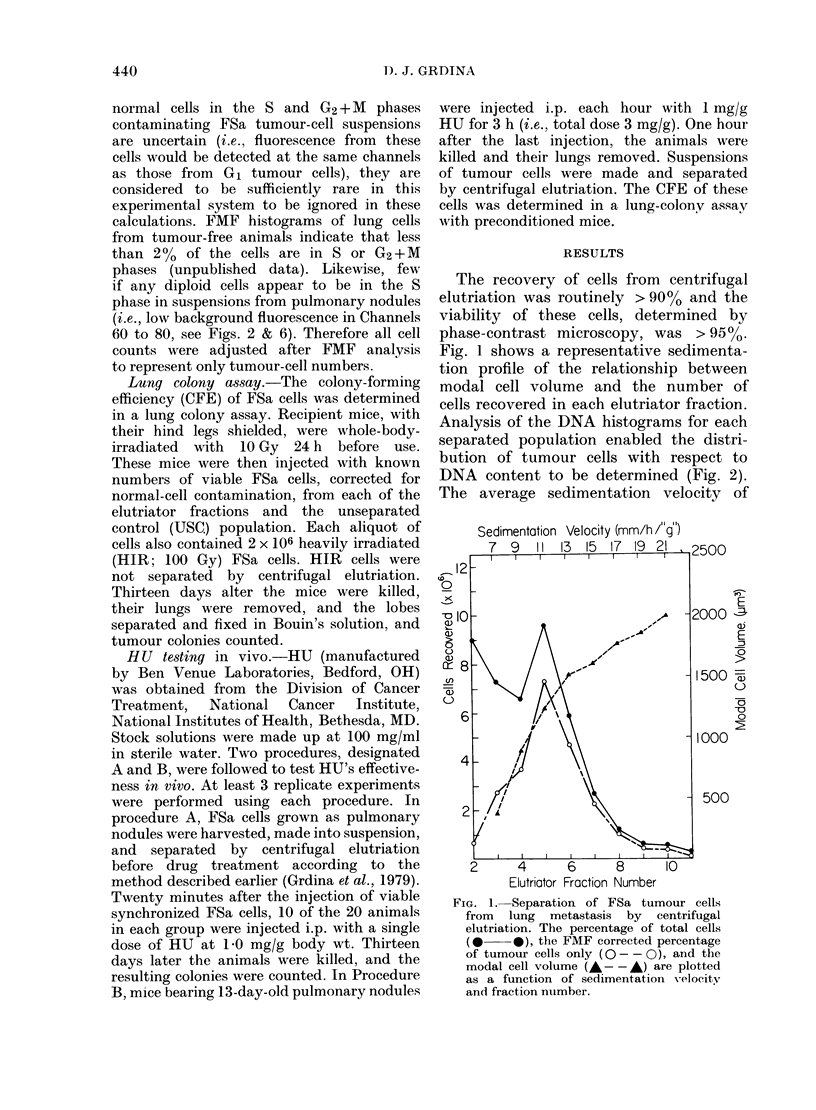

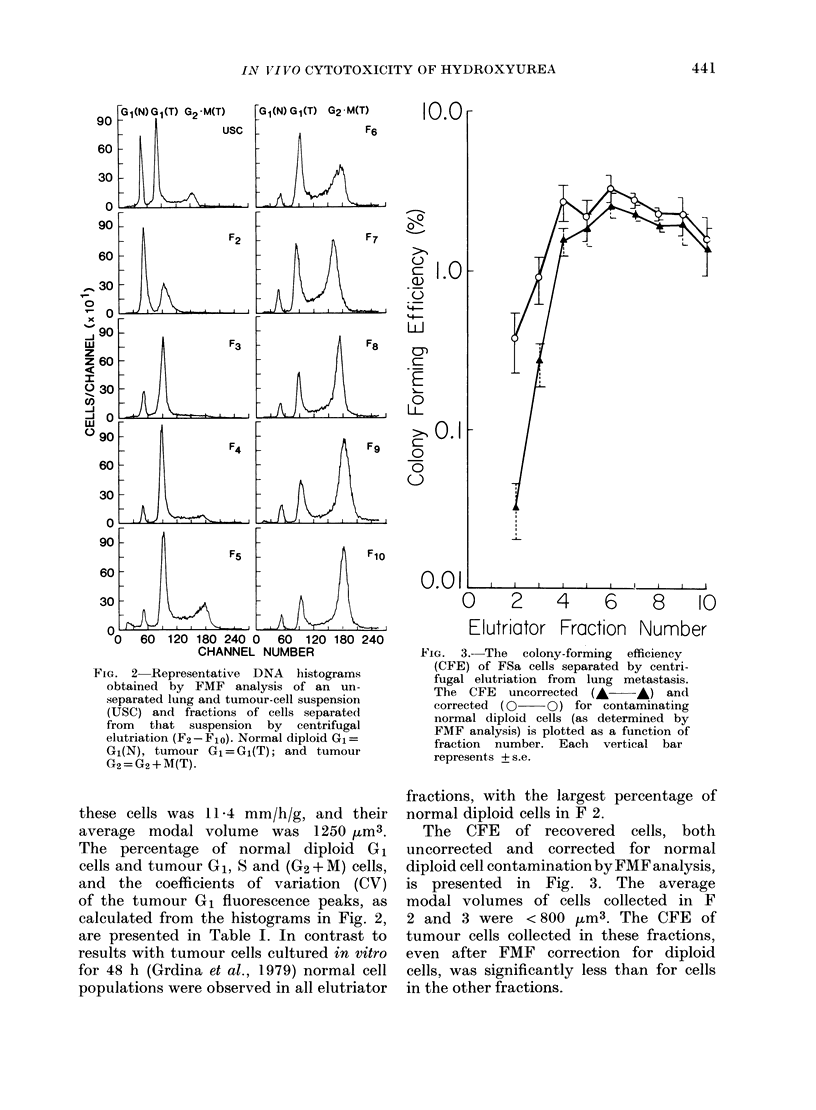

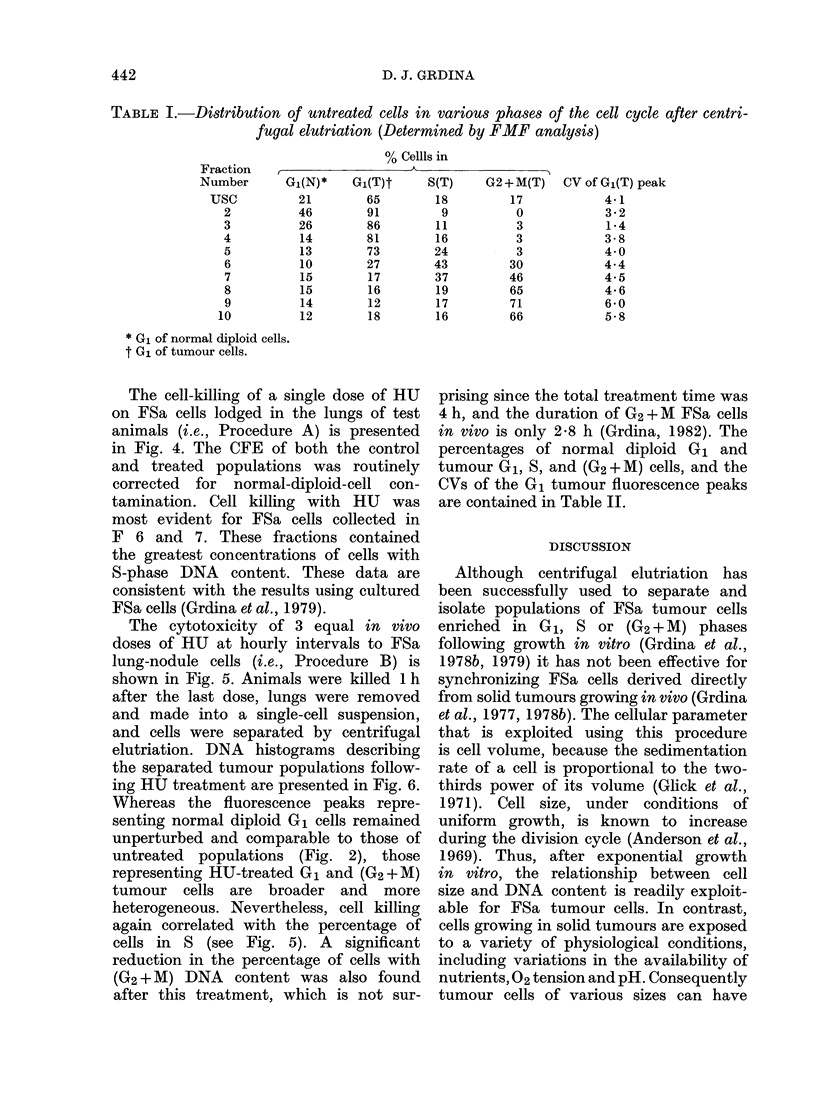

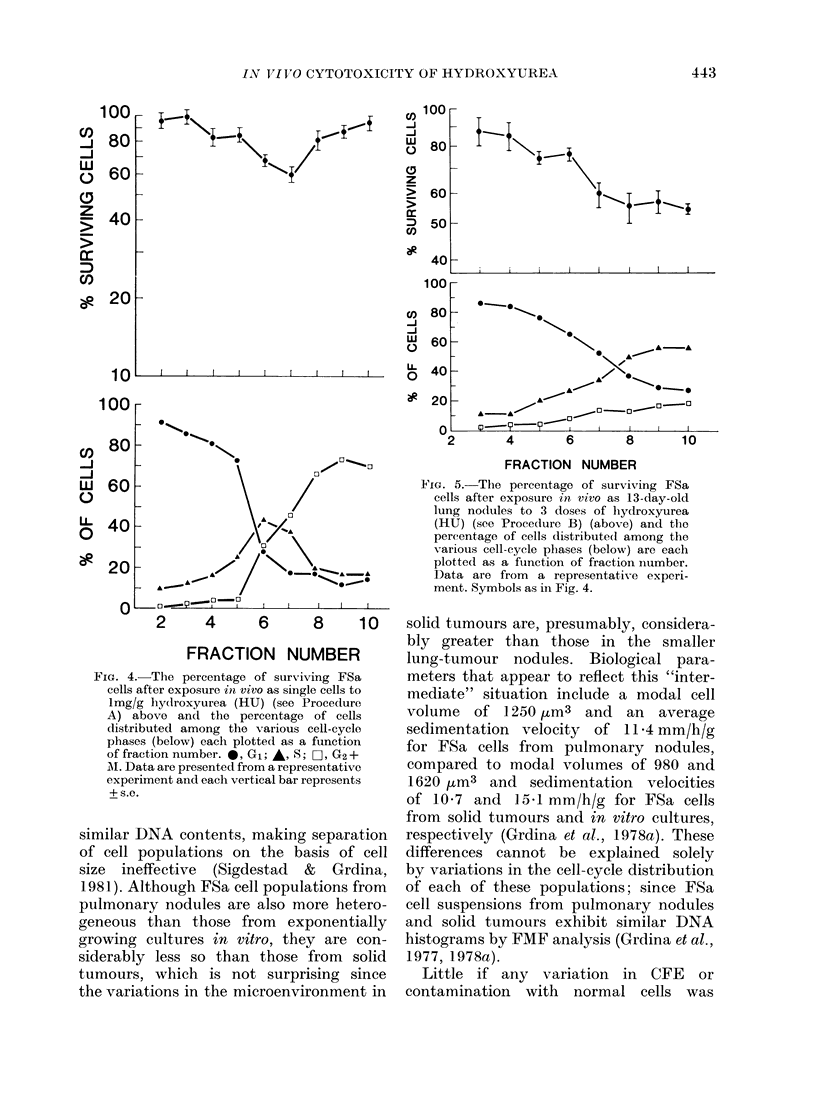

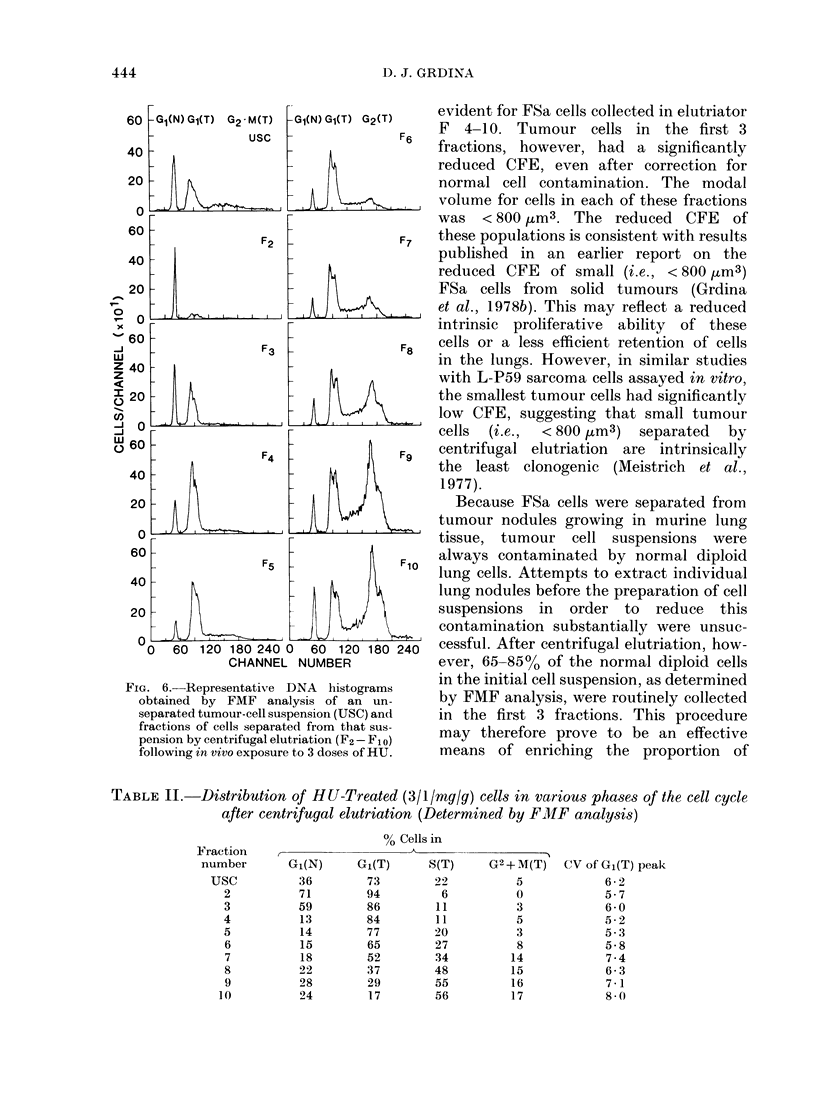

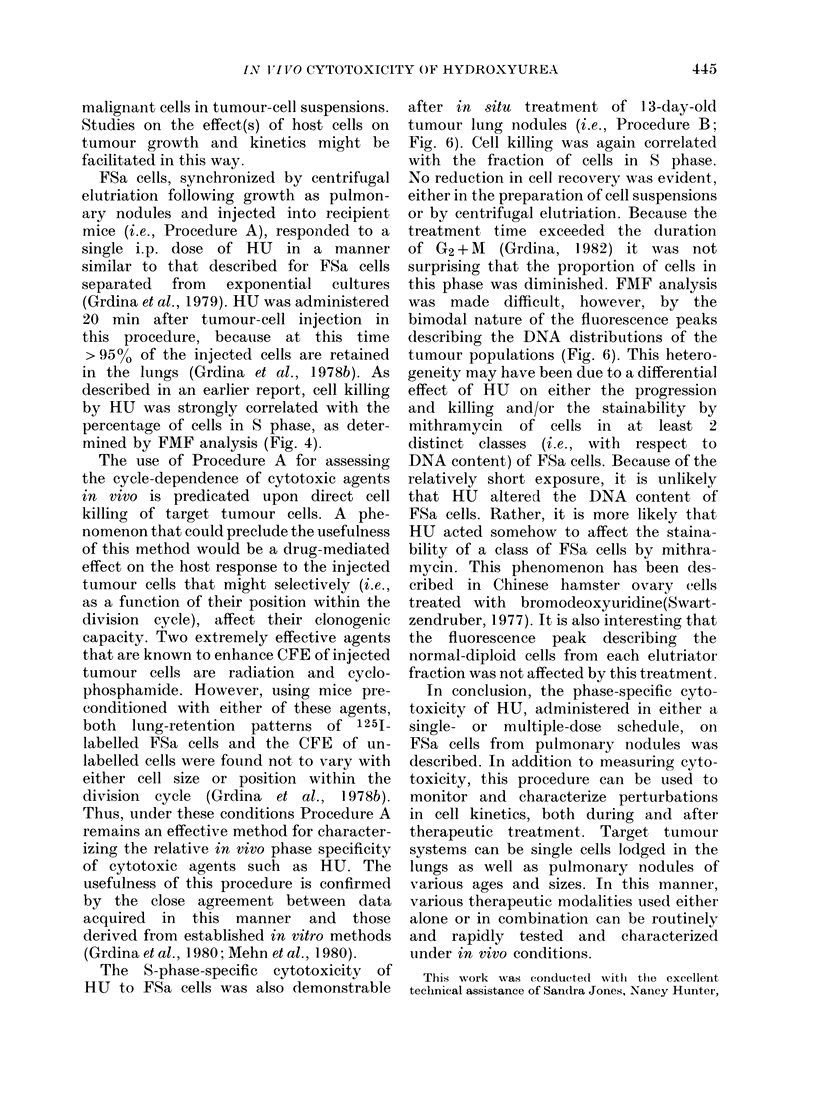

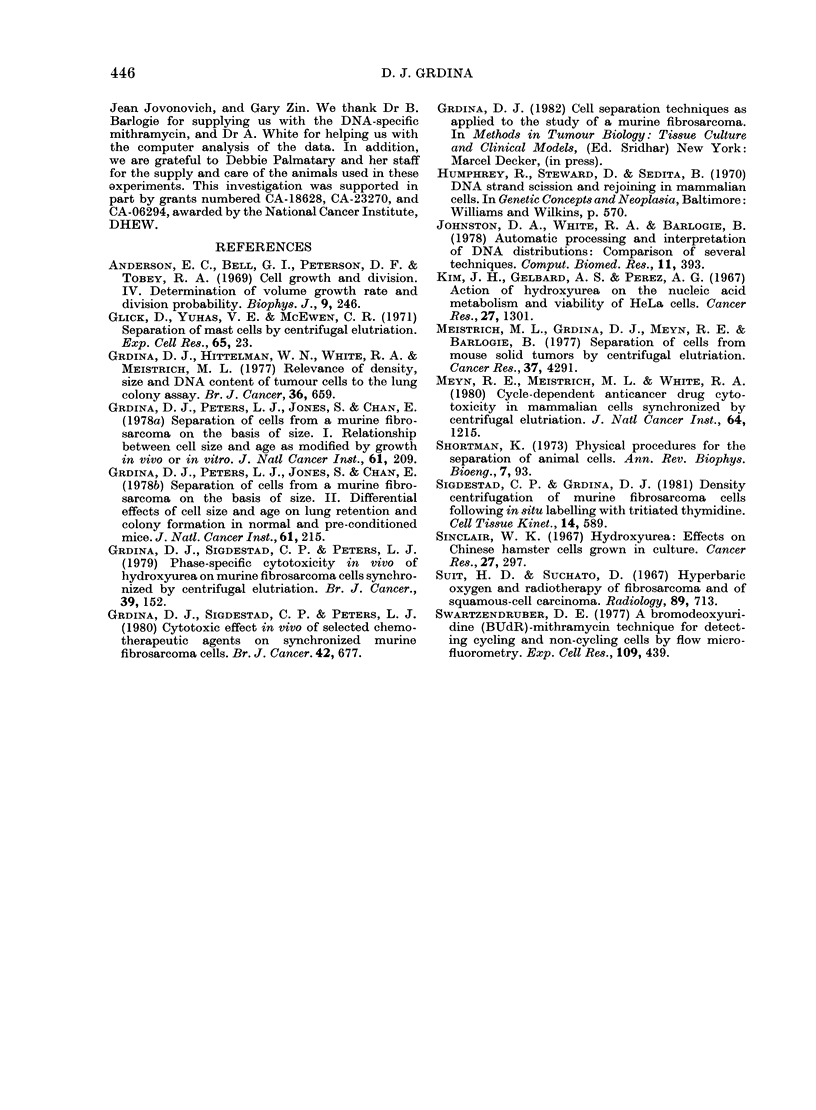


## References

[OCR_01042] Anderson E. C., Bell G. I., Petersen D. F., Tobey R. A. (1969). Cell growth and division. IV. Determination of volume growth rate and division probability.. Biophys J.

[OCR_01048] Glick D., Von Redlich D., Juhos E. T., McEwen C. R. (1971). Separation of mast cells by centrifugal elutriation.. Exp Cell Res.

[OCR_01053] Grdina D. J., Hittelman W. N., White R. A., Meistrich M. L. (1977). Relevance of density, size and DNA content of tumour cells to the lung colony assay.. Br J Cancer.

[OCR_01059] Grdina D. J., Peters L. J., Jones S., Chan E. (1978). Separation of cells from a murine fibrosarcoma on the basis of size. I. Relationship between cell size and age as modified by growth in vivo or in vitro.. J Natl Cancer Inst.

[OCR_01065] Grdina D. J., Peters L. J., Jones S., Chan E. (1978). Separation of cells from a murine fibrosarcoma on the basis of size. II. Differential effects of cell size and age on lung retention and colony formation in normal and preconditioned mice.. J Natl Cancer Inst.

[OCR_01080] Grdina D. J., Sigdestad C. P., Peters L. J. (1980). Cytotoxic effect in vivo of selected chemotherapeutic agents on synchronized murine fibrosarcoma cells.. Br J Cancer.

[OCR_01073] Grdina D. J., Sigdestad C. P., Peters L. J. (1979). Phase-specific cytotoxicity in vivo of hydroxyurea on murine fibrosarcoma cells synchronized by centrifugal elutriation.. Br J Cancer.

[OCR_01099] Johnston D. A., White R. A., Barlogie B. (1978). Automatic processing and interpretation of DNA distributions: comparison of several techniques.. Comput Biomed Res.

[OCR_01105] Kim J. H., Gelbard A. S., Perez A. G. (1967). Action of hydroxyurea on the nucleic acid metabolism and viability of HeLa cells.. Cancer Res.

[OCR_01111] Meistrich M. L., Grdina D. J., Meyn R. E., Barlogie B. (1977). Separation of cells from mouse solid tumors by centrifugal elutriation.. Cancer Res.

[OCR_01117] Meyn R. E., Meistrich M. L., White R. A. (1980). Cycle-dependent anticancer drug cytotoxicity in mammalian cells synchronized by centrifugal elutriation.. J Natl Cancer Inst.

[OCR_01129] Sigdestad C. P., Grdina D. J. (1981). Density centrifugation of murine fibrosarcoma cells following in situ labelling with tritiated thymidine.. Cell Tissue Kinet.

[OCR_01135] Sinclair W. K. (1967). Hydroxyurea: effects on Chinese hamster cells grown in culture.. Cancer Res.

[OCR_01140] Suit H. D., Suchato C. (1967). Hyperbaric oxygen and radiotherapy of a fibrosarcoma and of a squamous-cell carcinoma of C3H mice.. Radiology.

[OCR_01145] Swartzendruber D. E. (1977). A bromodeoxyuridine (BUdR)-mithramycin technique for detecting cycling and non-cycling cells by flow microfluorometry.. Exp Cell Res.

